# Effect of Neighborhood Deprivation Index on Breast Cancer Survival in the United States

**DOI:** 10.21203/rs.3.rs-2763010/v1

**Published:** 2023-04-07

**Authors:** Arya Mariam Roy, Anthony George, Kristopher Attwood, Sabah Alaklabi, Archit Patel, Angela R Omilian, Song Yao, Shipra Gandhi

**Affiliations:** Roswell Park Comprehensive Cancer Center; Roswell Park Comprehensive Cancer Center; Roswell Park Comprehensive Cancer Center; King Faisal Specialist Hospital and Research Center; Roswell Park Comprehensive Cancer Center; Roswell Park Comprehensive Cancer Center; Roswell Park Comprehensive Cancer Center; Roswell Park Comprehensive Cancer Center

**Keywords:** Socioeconomic status, neighborhood deprivation, breast cancer, health care disparities, early-stage breast cancer, overall survival

## Abstract

**Purpose:**

To analyze the association between the Neighborhood Deprivation Index (NDI) and clinical outcomes of early-stage breast cancer (BC).

**Methods:**

Surveillance, Epidemiology and End Results (SEER) database is queried to evaluate overall survival (OS) and disease-specific survival (DSS) of early- stage BC patients diagnosed between 2010–2016. Cox multivariate regression was performed to measure the association between NDI (Quintiles corresponding to most deprivation (Q1), above average deprivation (Q2), average deprivation (Q3), below average deprivation (Q4), least deprivation (Q5)) and OS/DSS.

**Results:**

Of the 88,572 early-stage BC patients, 27.4% (n = 24,307) were in the Q1 quintile, 26.5% (n = 23,447) were in the Q3 quintile, 17% (n = 15,035) were in the Q2 quintile, 13.5% (n = 11,945) were in the Q4 quintile, and 15.6% (n = 13,838) were in the Q5 quintile. There was a predominance of racial minorities in the Q1 and Q2 quintiles with Black women being 13–15% and Hispanic women being 15% compared to only 8% Black women and 6% Hispanic women in the Q5 quintile (p < 0.001). In multivariate analysis, in the overall cohort, those who live in Q2 and Q1 quintile have inferior OS and DSS compared to those who live in Q5 quintile (OS:- Q2: Hazard Ratio (HR) 1.28, Q1: HR 1.2; DSS:- Q2: HR 1.33, Q1: HR 1.25, all p < 0.001).

**Conclusion:**

Early-stage BC patients from areas with worse NDI have poor OS and DSS. Investments to improve the socioeconomic status of areas with high deprivation may help to reduce healthcare disparities and improve breast cancer outcomes.

## Introduction

Breast cancer (BC) is the most common malignancy in females globally and the most common cause of cancer-associated mortality [1]. Several demographics, clinicopathological factors including size, grade of the tumor, lymph node (LN) status, hormone receptor status, metastasis, age, and comorbidities of the patients are known to be associated with BC survival [2–4]. In addition to this, racial and ethnic backgrounds are also associated with breast cancer survival; Black women have 40% higher age-adjusted BC mortality than non-Hispanic White women[3,5]. The socioeconomic status (SES) of an individual and the neighborhood consists of multiple variables including income, education, occupation, living conditions which have been reported in relation to the survival of various cancers[6–8].

Reducing racial and ethnic and socioeconomic disparities in the access to health care have long been a major health policy goal in the United States (US).SES of the individual and neighborhood plays an important role in patient’s access to the health system[9]. Owing to the inequalities in opportunities, education, income, and developmental infrastructures, the areas with deprived individual and neighborhood SES may be associated with poor prognosis of certain malignancies and worse outcomes through multiple pathways[9,10]. Patients with poor individual SES may not be able to adhere to cancer screening guidelines, which happens due to their lack of awareness about the diseases, screening methods, lack of insurance, and occasionally the mistrust with the physicians/health care sector[11,12]. In addition to this, socioeconomically deprived neighborhoods lack proper healthcare resources, established referral systems, adequate social support, healthy lifestyle, and have transportation barriers to timely access to healthcare from the diagnosis to survivorship[10,13,14]. The myriad events rooting from poor SES affect cancer-related mortality and morbidity in vulnerable populations.

Although there were some studies done in the past evaluating the association of individual SES with the survival of cancer, epidemiological studies aiming at the association of SES and geographical variation in BC outcomes are limited. Given the fact that most of the factors owing to poor socioeconomic conditions are modifiable, it is very relevant to understand them and develop strategies to mitigate health disparities which aid us in improving health-related outcomes. In our study, we examine the association of neighborhood deprivation with the BC-related outcomes in patients with early-stage BC in the US.

## Methodology

### Data Sources

#### A. Neighborhood deprivation index

The neighborhood deprivation index (NDI) used in our analysis encompasses wealth and income, education, occupation, and housing conditions. Factor analysis was used to create the NDI for each census tract in the US, which identified key variables from 13 measures from the above dimensions proposed by Roux and Mair in their study assessing the contribution of neighborhood or residential environments to social and ethnic inequalities in health[15]. The key variables that are used from wealth and income are median household income, percent of household receiving dividends interest or rental income, percent of households receiving public assistance, median home value, percent of families with incomes below the poverty level. The variables from other dimensions are as follows: education (percent with a high school degree or higher; percent with a college degree or higher), occupation (percent in a management, business, science, or arts occupation; percent unemployed), and housing conditions (percent of households that are female-headed with any children under 18; percent of housing units that are owner occupied; percent of households without a telephone; percent of households without complete plumbing facilities)[16].NDI values range from ‐3.6 to +2.8 and higher values indicate more neighborhood deprivation which implies lower socioeconomic status. We used the NDI quintiles weighted by the tract population for the analysis. The first NDI quintile corresponds to most deprivation (Q1), second quintile (above average deprivation- Q2), third quintile (average deprivation- Q3), fourth quintile (below average deprivation (Q4)) and fifth quintile corresponds to least deprivation (Q5)[17].

#### B. Patient selection

We queried the Surveillance, Epidemiology and End Results (SEER) registry November 2021 submission database which covers approximately 48% of the US population for our study. We included early-stage BC pts (clinical stage group I, II, III), aged >=18 years, who are diagnosed from 2010–2016, and studied the overall survival (OS) and disease-specific survival (DSS) of BC in association with NDI. As data on HER2-neu status was accurately captured in SEER since 2010, this was chosen as the initial year of diagnosis for inclusion and selected patients diagnosed until 2016 to give adequate follow-up of 5 years. We excluded patients with unknown or missing data for each variable studied, or clinical/pathological evidence of distant metastases at the time of initial diagnosis. Institutional review board review was exempted as the data were deidentified and from publicly available databases upon request.

### Statistical Analysis.

The demographical and clinical characteristics of patients by NDI were tabulated by summary statistics. The mean, median, standard deviation, and range were used for continuous variables and the Kruskal-Wallis test was used for comparisons. For the categorical variables, frequencies and relative frequencies were compared using the chi-square test. The median, 3-year, 5-year OS and DSS were summarized by NDI using standard Kaplan-Meier methods.

Cox multivariate regression modeling was performed to test the association between NDI and OS, DSS, with adjustment for age, race, stage, grade, insurance status, surgery, radiation, and chemotherapy (CT). Subset analysis was done based on the BC subtypes (Estrogen receptor and/or progesterone receptor positive HER2-neu negative (HR+), HER2-neu-positive (HER2 +) and triple-negative breast cancer (TNBC). All statistics were performed using SAS software version 9.4 (SAS Institute Inc.) and significance testing was 2-sided at p<0.05. Data were analyzed from June 1, 2022 through July 15, 2022.

## Results

### Patient demographics

The baseline characteristics of the overall cohort are shown in Table 1.Of the 88,572 early-stage BC patients, 27.4 % (n= 24,307) were in the most deprivation (Q1) quintile, 26.5% (n= 23,447) were in the average deprivation (Q3) quintile, 17% (n= 15,035) were in the above average deprivation (Q2) quintile, 13.5% (n= 11,945) were in the below average deprivation (Q4) quintile, and 15.6% (n= 13,838) were in the least deprivation (Q5) quintile. The median age of patients in the Q5 quintile was 59 yrs and Q1 quintile was 61 yrs (p<0.001). There was a predominance of racial minorities in the Q1 and Q2 quintile with Black women being 13–15% and Hispanic women being 15% compared to only 8% Black women and 6% Hispanic women in the Q5 quintile (p<0.001). There was a higher percentage of uninsured patients in the Q1 quintile compared to the Q5 quintile (2.2% vs 1.7%, p<0.001). There were more rural areas in Q1 quintile compared to Q5 quintile (25.9% vs only 0.7%, p<0.001). There were more patients with stage III and grade III disease in Q1 quintile compared to Q5 quintile (Stage III: 28.7% vs 14.2%, Grade III: 34% vs 31.9%, p<0.001), and therefore, a greater percentage of patients received CT in Q1 quintile compared to Q5 quintile (44.6% vs 42.1%, p<0.001). However, fewer patients underwent surgery and radiation in the Q1 compared to the Q5 quintile, with 96.1% and 49.7% of patients undergoing surgery and radiation in Q1 quintile compared to 97.1 % and 56.5% in the Q5 quintile (p<0.001 for both).

There was a higher percentage of aggressive cancers such as TNBC and HER2+ BC in Q1 quintile compared to Q5 quintile (14.5%, 17.7% vs 11.7%, 16.5% respectively, p<0.001). The baseline characteristics were stratified by the subtype of breast cancer as shown in Table 2–4.It was observed that the patterns are similar in all the subtypes as observed in the overall cohort except that the patients who received chemotherapy for the early-stage BC were higher in the Q5 when compared to the Q1 in both TNBC and HER2+ BCs.

### Kaplan-Meier Survival Estimates

**Figure 1:**
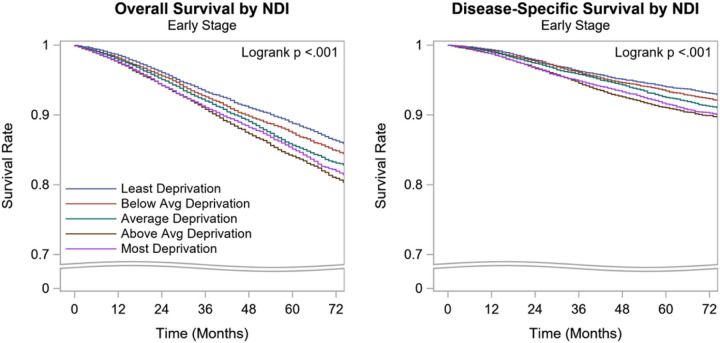
Kaplan-Meier Curves for overall and disease specific survival by NDI for early-stage breast cancer NDI: Neighborhood deprivation index, Avg: Average

On univariate analysis, after a median follow-up of 44 months, the 5-year OS rate of the overall cohort was 87%. The 5-year OS of the early-stage BC patients who live in the Q1 and Q2 quintile was low when compared to those who live in the Q5 quintile (85%, 84% vs 89%, p<0.001). The DSS of the overall cohort also followed a similar pattern (DSS of Q1, Q2 vsQ5: 92%, 91% vs 94%, p<0.001) (Table 5, Figure 1).

**Figure 2:**
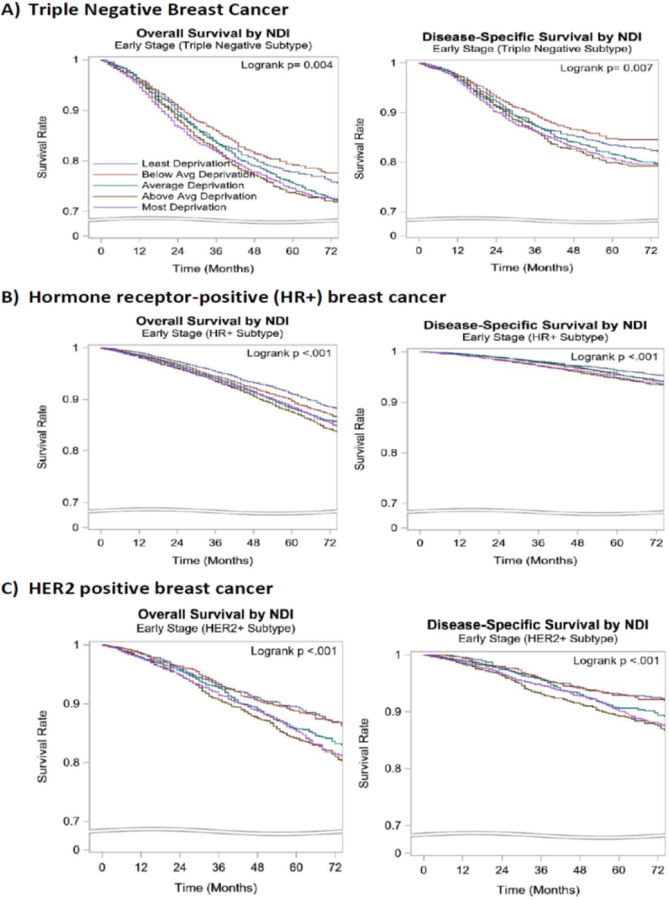
Kaplan-Meier Curves of overall and disease-specific survival by NDI for early-stage breast cancer subtypes NDI: Neighborhood deprivation index, Avg: Average, N: Number, HR+ : Hormone receptor-positive human epidermal growth factor 2- negative, HER2 + : Human epidermal growth factor receptor 2 positive

In subset analysis stratified by the BC subtypes, the 5-year OS and DSS were lower in the Q1 and Q2 quintiles compared to the Q5 quintile in all the subtypes of BC (HR+, HER2 + and TNBC). However, the 5-year DSS rate was not significantly different in the HR+ subtype (Q1, Q2 vs Q5: 95%, 95% vs 96%, p<0.001). (Table 5, Figure 2).

### Multivariate Survival Analysis

**Figure 3:**
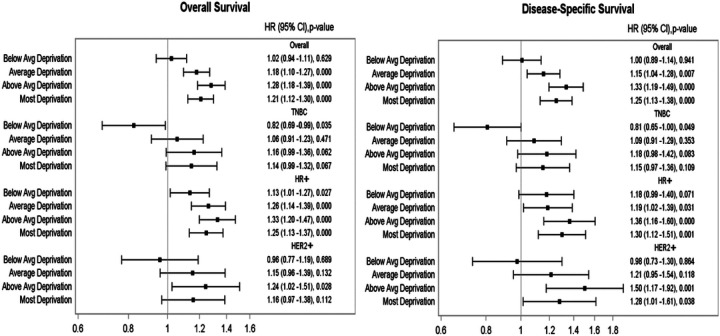
Multivariate survival analysis in the early-stage overall cohort: overall survival and disease-specific survival :- HR (95% CI): Hazard ratio (95% confidence interval), Avg: Average, TNBC: Triple negative breast cancer, HR+: Hormone receptor-positive human epidermal growth factor 2- negative, HER2+ : Human epidermal growth factor receptor 2 positive

In multivariate analysis after adjusting for socio-demographic, clinical, and treatment variables, in the overall cohort, those who live in Q2 quintile and Q1 quintile have inferior OS and DSS when compared to those who live in Q5 quintile (OS in Q2: Hazard Ratio (HR) 1.28; OS in Q1: HR 1.2; DSS in Q2: HR 1.33; DSS in Q1: HR 1.25, all p<0.001). In the subset analysis, similar results for OS and DSS by NDI were observed in hormone receptor positive HER2 negative (HR+) and HER2+ subtypes, but not in TNBC (Figure 3).

## Discussion

Our study focuses on early-stage BC as its treatment requires access to health care systems that are less available in poor resource neighborhoods. In our study, we found that the deprivation index of the neighborhoods was in significant association with BC survival. Our analysis showed that the OS and DSS of patients with early-stage BC were lower for those who live in socioeconomically deprived neighborhoods compared to those who live in affluent neighborhoods. The survival differences were observed among all subtypes of BC. The differences in survival persisted even after adjusting for demographic, clinical, and treatment factors that could affect breast cancer survival. On multivariate analysis, the mortality difference among the patients living in different socioeconomic areas was statistically significant within the overall cohort, HR+ and HER2+ BC subtypes, but not within the TNBC subtype. This could be explained by the aggressive nature of TNBC. As TNBC is very aggressive and has high relapse rate[18], the survival of patients with TNBC could be poor regardless of their socioeconomic status.

Understanding the impact of neighborhood deprivation on BC survival will facilitate the development and implementation of policies and prioritize investments in communities with high deprivation scores. This could improve the socioeconomic conditions which could eventually improve clinical outcomes.[19] Several factors in the neighborhood influence the health of an individual directly, as well as indirectly: poverty, access to the health care system, transportation system, housing quality, unemployment, environmental pollution including air and water pollution, neighborhood hygiene, waste management system, crime rates, racial composition, educational system, tobacco availability and marketing, access to healthy food[20–23]. These along with the factors that affect the individual such as marital status, family/social support, co-morbidities, mental health, nutritional status, healthy lifestyle, insurance status, and educational status play an inevitable role in the survival outcomes of malignancies. Studies have shown that prolonged and cumulative exposure to the above-mentioned deprivation-associated stressors can induce chronic inflammation which is one of the etiologies behind cancer development[24,25].Therefore, a proper understanding of the deprivation factors of an individual and their neighborhood is essential to plan interventions to reduce the burden of cancer mortality.

Our study adds to the existing literature in multiple ways. This study is the first to our knowledge to use a national database to examine the association between neighborhood deprivation with the clinical outcomes of early-stage BC; most prior studies used regional databases. Prior studies showed racial disparities in BC-related outcomes in the US and minoritized groups tend to have higher BC-related mortality[22]. In a study by Luningham et. al. which examined the association between racial disparities and SES in BC survival between Black and White women across Georgia, it was found that Black women with BC had higher mortality than White women, but this disparity was not explained by the socioeconomic deprivation of their residential area. White patients living in socioeconomically affluent areas had lower rates of BC mortality compared to those who reside in deprived neighborhoods [26]. This study finding critically shows the important role of area of residence on clinical outcomes, and thus emphasizing that socioeconomic factors of the neighborhood play a vital role in determining clinical outcomes. There were several studies conducted to understand the reason behind the observed racial disparities. One of them was attributed to poor neighborhoods. Black and Hispanic women generally live in poor neighborhoods and Black patients were found to live in neighborhoods with high poverty rates and this difference persists even after adjusting for their income status[27]. In our study, we found that Black and Hispanic women with BC were more commonly residing in the deprived neighborhoods compared to socioeconomically affluent neighborhoods; however, the disparities in BC-related mortality of the patients from these socioeconomically different neighborhoods persisted even after accounting for the racial disparities.

Similarly, uninsured patients, rural areas were found more commonly in the deprived neighborhoods. Advanced BCs (higher stage and grade) and aggressive BC such as HER2 + and TNBC were predominantly found in the deprived neighborhoods compared to the affluent neighborhoods. In our study, BC patients from affluent neighborhoods received more surgical and radiation treatments which can be explained by the higher percentages of urban areas in these regions with better facilities for treatments, and better referral systems. Our study showed that in the overall BC cohort, patients who received chemotherapy were slightly higher in the deprived neighborhoods than in the affluent neighborhood. One possible explanation for this is that the advanced diseases that require chemotherapy were more prevalent in the deprived neighborhood regions. Nevertheless, the disparities in BC-related mortality remain unaffected when adjusted for the demographic, clinical, pathological, and treatment-related factors such as age, race, stage, grade, insurance, surgery, radiation, and chemotherapy. This suggests that additional factors related to neighborhood SES that are not captured by the NDI play an important role in BC-related outcomes. The access to genetic and somatic testing which are important for deciding the appropriate treatments in BC might be limited to patients from poor neighborhood which could have impacted their survival.

Poverty, unhealthy food habits, decreased access to healthy food, environmental pollution, increased advertising of tobacco in poor neighborhood leads to increased incidence of various cancers in patients from these neighborhoods[20,21,23,28,29]. Furthermore, the transportation barriers, decreased access to better comprehensive cancer centers with standard of care treatments or novel clinical trials, poor nutritional and educational status of patients, financial toxicity associated with cancer treatment leads to increased cancer-related mortality in socioeconomically poor neighborhoods[10,14,30,31]. In addition to this, poor environmental hygiene and pollution can add to the increased mortality by causing infections in cancer patients who are already immunocompromised due to cancer and associated treatments.[32] In a patient-reported outcomes study in advanced cancers, it was found that patients from socioeconomically deprived areas have a higher level of anxiety.[33] Factors such as anxiety, depression, and poor social support which are subjective measures of poor mental health are not accounted for in any of the tools to measure the neighborhood/individual SES and have been shown to be associated with cancer-related mortality.[13,33,34] Disparities in BC survival related to neighborhood SES reflect the systematic barriers in policies related to health care, education, employment, insurance, environment, and judicial system. Our study findings may support restructuring the policies, to implement new policies and investments in socioeconomically deprived neighborhoods which would help to decrease inequalities in opportunities, improve healthcare facilities, and increase access to timely cancer treatments.

Our study has many strengths and certain limitations. We used large real-world data to assess the impact of neighborhood deprivation on clinical outcomes of BC patients. These data capture more than 50% of the US population, and therefore, the results are generalizable. We adjusted for multiple factors that are known to influence survival, including racial distribution, demographic factors, clinicopathological factors of the disease [35]. Although NDI is a comprehensive tool to assess socioeconomic disadvantage, it may not capture all the factors associated with neighborhood SES. We could not assess the influence of several neighborhood factors that may contribute to the mortality of BC such as access to transportation, environmental: air, water pollution, poverty level, accessibility to healthy food, and crime rate of the neighborhood. As we do not have one comprehensive tool to assess the socioeconomic status of neighborhoods and individuals together, future studies warranting the combination of multiple indices such as area deprivation index, Yost index might be beneficial. As it is a retrospective national database study, several individual factors that can affect the mortality rates of BC such as comorbidities of patients, social support, details of factors such as anxiety, and depression that can affect the mental and physical condition of the patients were not collected. Incompleteness of individual-level data collected on cancer risk and treatment, and incomplete values for several variables collected from multiple registries were other limitations. Further tumor recurrence data, specific details on the type, dose, and duration of chemotherapy, radiation, oral chemotherapy, targeted agents were not available in the SEER database and these factors could have been associated with the mortality of BC.

## Conclusion

The findings from our study suggest that neighborhood deprivation is significantly and independently associated with worse clinical outcomes among patients with BC in the US. Early-stage BC patients from areas with worse NDI have poor OS and DSS, after accounting for demographic, clinicopathological, and treatment-related factors. Identification of these poor-resource neighborhoods is critical to guide investments in these neighborhoods and implement policies focusing on improving the SES of these areas with high deprivation to reduce healthcare disparities and improve breast cancer outcomes. Future studies are warranted to understand the factors affecting the neighborhood socioeconomic status other than what is mentioned in our study and to assess their relationship with BC-related survival. The data from these studies might be extrapolated to other cancers which would help us to improve the quality of life of patients and cancer-related mortalities.

## Figures and Tables

**Table 1:** Baseline characteristics by NDI in the early-stage breast cancer overall cohort

		Least Deprivation	Below Avg Deprivation	Average Deprivation	Above Avg Deprivation	Most Deprivation	P-value
	N	13,838 (15.6)	11,945 (13.5)	23,447 (26.5)	15,035 (17.0)	24,307 (27.4)	
Age	Mean/Std/N	59.77/13.33/13838	60.55/13.33/11945	60.57/13.24/23447	60.97/13.16/15035	60.57/13.18/24307	<.00
	Median/Min/Max	59.00/20.00/102.00	60.00/19.00/100.00	61.00/17.00/102.00	61.00/19.00/108.00	61.00/20.00/102.00	
Sex	Female	13,731 (99.2%)	11,866 (99.3%)	23,265 (99.2%)	14,924 (99.3%)	24,156 (99.4%)	0.23
	Male	107 (0.8%)	79 (0.7%)	182 (0.8%)	111 (0.7%)	151 (0.6%)	
Race	White	11,628 (84.0%)	10,260 (85.9%)	19,746 (84.2%)	12,315 (81.9%)	20,124 (82.8%)	<.00
	Black	1,136 (8.2%)	850 (7.1%)	2,657 (11.3%)	2,293 (15.3%)	3,147 (12.9%)	
	Other	1,074 (7.8%)	835 (7.0%)	1,044 (4.5%)	427 (2.8%)	1,036 (4.3%)	
Hispanic	No	13,013 (94.0%)	10,726 (89.8%)	21,892 (93.4%)	14,276 (95.0%)	20,718 (85.2%)	<.00
	Yes	825 (6.0%)	1,219 (10.2%)	1,555 (6.6%)	759 (5.0%)	3,589 (14.8%)	
Insurance	Insured	13,597 (98.3%)	11,693 (97.9%)	23,064 (98.4%)	14,725 (97.9%)	23,769 (97.8%)	<.00
	Uninsured	241 (1.7%)	252 (2.1%)	383 (1.6%)	310 (2.1%)	538 (2.2%)	
Location	Urban	13,736 (99.3%)	10,395 (87.0%)	17,517 (74.7%)	8,375 (55.7%)	18,012 (74.1%)	<.00
	Rural	102 (0.7%)	1,550 (13.0%)	5,930 (25.3%)	6,660 (44.3%)	6,295 (25.9%)	
Grade	I/II	8,962 (64.8%)	7,585 (63.5%)	14,914 (63.6%)	9,318 (62.0%)	15,091 (62.1%)	<.01
	III	4,409 (31.9%)	3,791 (31.7%)	7,728 (33.0%)	5,117 (34.0%)	8,271 (34.0%)	
	Unknown	467 (3.4%)	569 (4.8%)	805 (3.4%)	600 (4.0%)	945 (3.9%)	
Stage	I/II	12,266 (15.8)	10,507 (13.6)	20,608 (26.6)	12,968 (16.7)	21,122 (27.3)	<0.0
	III	1,572 (14.2)	1,438 (13.0)	2,839 (25.6)	2,067 (18.6)	3,185 (28.7)	
Lateral	Left	6,941 (50.2%)	6,025 (50.4%)	11,940 (50.9%)	7,747 (51.5%)	12,343 (50.8%)	0.18
	Right	6,897 (49.8%)	5,920 (49.6%)	11,507 (49.1%)	7,288 (48.5%)	11,964 (49.2%)	
Surgery	Yes	13,439 (97.1%)	11,440 (95.8%)	22,601 (96.4%)	14,501 (96.4%)	23,358 (96.1%)	<.00
	No	393 (2.8%)	496 (4.2%)	831 (3.5%)	504 (3.4%)	904 (3.7%)	
	Unknown	6 (0.0%)	9 (0.1%)	15 (0.1%)	30 (0.2%)	45 (0.2%)	
Radiation	Yes	7,821 (56.5%)	6,373 (53.4%)	12,731 (54.3%)	8,019 (53.3%)	12,076 (49.7%)	<.00
	No	5,971 (43.1%)	5,495 (46.0%)	10,603 (45.2%)	6,908 (45.9%)	12,121 (49.9%)	
	Total	46 (0.3%)	77 (0.6%)	113 (0.5%)	108 (0.7%)	110 (0.5%)	
Chemo therapy	Yes	5,825 (42.1%)	4,962 (41.5%)	9,970 (42.5%)	6,750 (44.9%)	10,853 (44.6%)	<.00
	None/Unknown	8,013 (57.9%)	6,983 (58.5%)	13,477 (57.5%)	8,285 (55.1%)	13,454 (55.4%)	
Subtype	Triple negative	1,390 (11.7%)	1,280 (12.7%)	2,653 (13.3%)	1,843 (14.5%)	2,960 (14.5%)	<.00
	HR+	8,511 (71.8%)	7,113 (70.6%)	13,994 (70.3%)	8,640 (68.0%)	13,850 (67.8%)	
	HER2+	1,956 (16.5%)	1,689 (16.8%)	3,264 (16.4%)	2,225 (17.5%)	3,625 (17.7%)	

Table 1: NDI- Neighborhood deprivation index, Avg: Average, N: Number, HR+ : Hormone receptor-positive human epidermal growth factor 2- negative, HER2 + : Human epidermal growth factor receptor 2 positive

**Table 2:** Baseline characteristics by NDI in early-stage triple negative breast cancer subtypes

		Least Deprivation	Below Avg Deprivation	Average Deprivation	Above Avg Deprivation	Most Deprivation	P-value
	N	1,390 (13.7)	1,280 (12.6)	2,653 (26.2)	1,843 (18.2)	2,960 (29.2)	
Age	Mean/Std/N	56.76/13.52/1390	57.92/13.82/1280	57.98/13.73/2653	57.26/13.62/1843	57.36/13.70/2960	0.0
	Median/Min/Max	56.00/24.00/102.00	57.00/24.00/96.00	58.00/22.00/100.00	57.00/24.00/99.00	57.00/24.00/96.00	
Sex	Female	1,389 (99.9%)	1,280 (100.0%)	2,651 (99.9%)	1,839 (99.8%)	2,957 (99.9%)	0.3
	Male	1 (0.1%)	0	2 (0.1%)	4 (0.2%)	3 (0.1%)	
Race	White	1,069 (76.9%)	1,006 (78.6%)	2,031 (76.6%)	1,320 (71.6%)	2,198 (74.3%)	<0
	Black	216 (15.5%)	186 (14.5%)	502 (18.9%)	483 (26.2%)	670 (22.6%)	
	Other	105 (7.6%)	88 (6.9%)	120 (4.5%)	40 (2.2%)	92 (3.1%)	
Hispanic	No	1,285 (92.4%)	1,125 (87.9%)	2,445 (92.2%)	1,742 (94.5%)	2,501 (84.5%)	<0
	Yes	105 (7.6%)	155 (12.1%)	208 (7.8%)	101 (5.5%)	459 (15.5%)	
Insurance	Insured	1,354 (97.4%)	1,252 (97.8%)	2,590 (97.6%)	1,785 (96.9%)	2,865 (96.8%)	0.1
	Uninsured	36 (2.6%)	28 (2.2%)	63 (2.4%)	58 (3.1%)	95 (3.2%)	
Location	Urban	1,379 (99.2%)	1,126 (88.0%)	2,037 (76.8%)	1,023 (55.5%)	2,163 (73.1%)	<0
	Rural	11 (0.8%)	154 (12.0%)	616 (23.2%)	820 (44.5%)	797 (26.9%)	
Grade	I/II	225 (16.2%)	221 (17.3%)	450 (17.0%)	331 (18.0%)	520 (17.6%)	0.0
	III	1,135 (81.7%)	1,011 (79.0%)	2,127 (80.2%)	1,446 (78.5%)	2,328 (78.6%)	
	Unknown	30 (2.2%)	48 (3.8%)	76 (2.9%)	66 (3.6%)	112 (3.8%)	
Stage	I/II	1,184 (14.1%)	1,080 (12.8%)	2,211 (26.3%)	1,511 (17.9%)	2,436 (28.9%)	0.0
	III	206 (12.1%)	200 (11.7%)	442 (25.9%)	332 (19.5%)	524 (30.8%)	
Lateral	Left	681 (49.0%)	655 (51.2%)	1,343 (50.6%)	949 (51.5%)	1,521 (51.4%)	0.6
	Right	709 (51.0%)	625 (48.8%)	1,310 (49.4%)	894 (48.5%)	1,439 (48.6%)	
Surgery	Yes	1,340 (96.4%)	1,208 (94.4%)	2,534 (95.5%)	1,779 (96.5%)	2,822 (95.3%)	0.0
	No	50 (3.6%)	71 (5.5%)	114 (4.3%)	56 (3.0%)	133 (4.5%)	
	Unknown	0	1 (0.1%)	5 (0.2%)	8 (0.4%)	5 (0.2%)	
Radiation	Yes	737 (53.0%)	652 (50.9%)	1,369 (51.6%)	1,011 (54.9%)	1,482 (50.1%)	0.0
	No	649 (46.7%)	623 (48.7%)	1,271 (47.9%)	823 (44.7%)	1,468 (49.6%)	
	Total	4 (0.3%)	5 (0.4%)	13 (0.5%)	9 (0.5%)	10 (0.3%)	
Chemotherapy	Yes	1,087 (78.2%)	945 (73.8%)	2,042 (77.0%)	1,425 (77.3%)	2,293 (77.5%)	0.0
	None/Unknown	303 (21.8%)	335 (26.2%)	611 (23.0%)	418 (22.7%)	667 (22.5%)	
Subtype	Triple negative	1,390 (100.0%)	1,280 (100.0%)	2,653 (100.0%)	1,843 (100.0%)	2,960 (100.0%)	<0

Table 2: NDI- Neighborhood deprivation index, Avg: Average, N: Number, HR+ : Hormone receptor-positive human epidermal growth factor 2- negative, HER2 + : Human epidermal growth factor receptor 2 positive

**Table 3:** Baseline characteristics by NDI in early-stage hormone receptor positive (HR+) subtype

		Least Deprivation	Below Avg Deprivation	Average Deprivation	Above Avg Deprivation	Most Deprivation
1	N	8,511 (16.3)	7,113 (13.7)	13,994 (26.9)	8,640 (16.6)	13,850 (26.6)
Age	Mean/Std/N	60.80/13.01/8511	61.37/13.02/7113	61.46/12.91/13994	61.89/12.73/8640	61.64/12.84/13850
	Median/Min/Max	61.00/24.00/101.00	62.00/19.00/100.00	62.00/22.00/97.00	62.00/19.00/100.00	62.00/21.00/102.00
Sex	Female	8,438 (99.1%)	7,060 (99.3%)	13,867 (99.1%)	8,567 (99.2%)	13,736 (99.2%)
	Male	73 (0.9%)	53 (0.7%)	127 (0.9%)	73 (0.8%)	114 (0.8%)
Race	White	7,339 (86.2%)	6,246 (87.8%)	12,178 (87.0%)	7,309 (84.6%)	11,888 (85.8%)
	Black	541 (6.4%)	381 (5.4%)	1,229 (8.8%)	1,079 (12.5%)	1,399 (10.1%)
	Other	631 (7.4%)	486 (6.8%)	587 (4.2%)	252 (2.9%)	563 (4.1%)
Hispanic	No	8,046 (94.5%)	6,449 (90.7%)	13,123 (93.8%)	8,231 (95.3%)	11,880 (85.8%)
	Yes	465 (5.5%)	664 (9.3%)	871 (6.2%)	409 (4.7%)	1,970 (14.2%)
Insurance	Insured	8,385 (98.5%)	6,962 (97.9%)	13,822 (98.8%)	8,496 (98.3%)	13,599 (98.2%)
	Uninsured	126 (1.5%)	151 (2.1%)	172 (1.2%)	144 (1.7%)	251 (1.8%)
Location	Urban	8,454 (99.3%)	6,177 (86.8%)	10,381 (74.2%)	4,873 (56.4%)	10,340 (74.7%)
	Rural	57 (0.7%)	936 (13.2%)	3,613 (25.8%)	3,767 (43.6%)	3,510 (25.3%)
Grade	I/II	6,814 (80.1%)	5,537 (77.8%)	11,177 (79.9%)	6,786 (78.5%)	10,840 (78.3%)
	III	1,491 (17.5%)	1,279 (18.0%)	2,468 (17.6%)	1,582 (18.3%)	2,591 (18.7%)
	Unknown	206 (2.4%)	297 (4.2%)	349 (2.5%)	272 (3.1%)	419 (3.0%)
Stage	I/II	7,724 (16.5%)	6,415 (13.7%)	12,626 (26.9%)	7,689 (16.4%)	12,443 (26.5%)
	III	787 (15.1%)	698 (13.4)	1,368 (26.3%)	951 (18.3%)	1,407 (27.0)
Lateral	Left	4,293 (50.4%)	3,552 (49.9%)	7,075 (50.6%)	4,410 (51.0%)	7,002 (50.6%)
	Right	4,218 (49.6%)	3,561 (50.1%)	6,919 (49.4%)	4,230 (49.0%)	6,848 (49.4%)
Surgery	Yes	8,317 (97.7%)	6,885 (96.8%)	13,605 (97.2%)	8,396 (97.2%)	13,439 (97.0%)
	No	190 (2.2%)	222 (3.1%)	384 (2.7%)	236 (2.7%)	389 (2.8%)
	Unknown	4 (0.0%)	6 (0.1%)	5 (0.0%)	8 (0.1%)	22 (0.2%)
Radiation	Yes	5,020 (59.0%)	4,018 (56.5%)	7,961 (56.9%)	4,786 (55.4%)	7,234 (52.2%)
	No	3,460 (40.7%)	3,040 (42.7%)	5,955 (42.6%)	3,787 (43.8%)	6,555 (47.3%)
	Total	31 (0.4%)	55 (0.8%)	78 (0.6%)	67 (0.8%)	61 (0.4%)
Chemotherapy	Yes	2,494 (29.3%)	2,064 (29.0%)	4,023 (28.7%)	2,710 (31.4%)	4,277 (30.9%)
	None/Unknown	6,017 (70.7%)	5,049 (71.0%)	9,971 (71.3%)	5,930 (68.6%)	9,573 (69.1%)
Subtype	HR+	8,511 (100.0%)	7,113 (100.0%)	13,994 (100.0%)	8,640 (100.0%)	13,850 (100.0%)

Table 3: NDI- Neighborhood deprivation index, Avg: Average, N: Number, HR+ : Hormone receptor-positive human epidermal growth factor 2- negative, HER2 + : Human epidermal growth factor receptor 2 positive

**Table 4:** Baseline characteristics by NDI in early-stage HER2-positive (HER2+) subtype

		Least Deprivation	Below Avg Deprivation	Average Deprivation	Above Avg Deprivation	Most Deprivation	P-value
	N	1,956 (15.3)	1,689 (13.2)	3,264 (25.6)	2,225 (17.4)	3,625 (28.4)	
Age	Mean/Std/N	55.62/13.16/1956	56.91/13.38/1689	57.32/13.42/3264	58.74/13.39/2225	57.76/13.15/3625	<0.001
	Median/Min/Max	54.00/20.00/96.00	56.00/20.00/96.00	57.00/17.00/102.00	58.00/24.00/94.00	57.00/20.00/100.00	
Sex	Female	1,941 (99.2%)	1,679 (99.4%)	3,233 (99.1%)	2,212 (99.4%)	3,615 (99.7%)	0.009
	Male	15 (0.8%)	10 (0.6%)	31 (0.9%)	13 (0.6%)	10 (0.3%)	
Race	White	1,591 (81.3%)	1,403 (83.1%)	2,633 (80.7%)	1,777 (79.9%)	2,890 (79.7%)	<0.001
	Black	175 (8.9%)	138 (8.2%)	444 (13.6%)	375 (16.9%)	548 (15.1%)	
	Other	190 (9.7%)	148 (8.8%)	187 (5.7%)	73 (3.3%)	187 (5.2%)	
Hispanic	No	1,821 (93.1%)	1,490 (88.2%)	3,005 (92.1%)	2,101 (94.4%)	3,023 (83.4%)	<0.001
	Yes	135 (6.9%)	199 (11.8%)	259 (7.9%)	124 (5.6%)	602 (16.6%)	
Insurance	Insured	1,912 (97.8%)	1,644 (97.3%)	3,175 (97.3%)	2,164 (97.3%)	3,534 (97.5%)	0.831
	Uninsured	44 (2.2%)	45 (2.7%)	89 (2.7%)	61 (2.7%)	91 (2.5%)	
Location	Urban	1,944 (99.4%)	1,484 (87.9%)	2,521 (77.2%)	1,237 (55.6%)	2,680 (73.9%)	<0.001
	Rural	12 (0.6%)	205 (12.1%)	743 (22.8%)	988 (44.4%)	945 (26.1%)	
Grade	I/II	730 (37.3%)	696 (41.2%)	1,247 (38.2%)	879 (39.5%)	1,452 (40.1%)	0.048
	III	1,146 (58.6%)	901 (53.3%)	1,872 (57.4%)	1,246 (56.0%)	2,024 (55.8%)	
	Unknown	80 (4.1%)	92 (5.4%)	145 (4.4%)	100 (4.5%)	149 (4.1%)	
Stage	I/II	1,616 (15.7%)	1,377 (13.3%)	2,638 (25.6%)	1,766 (17.1%)	2,926 (28.3%)	0.1063
	III	340 (14.0%)	312 (12.8%)	626 (25.7%)	459 (18.8%)	699 (28.7%)	
Lateral	Left	1,001 (51.2%)	851 (50.4%)	1,673 (51.3%)	1,166 (52.4%)	1,837 (50.7%)	0.710
	Right	955 (48.8%)	838 (49.6%)	1,591 (48.7%)	1,059 (47.6%)	1,788 (49.3%)	
Surgery	Yes	1,876 (95.9%)	1,599 (94.7%)	3,112 (95.3%)	2,136 (96.0%)	3,417 (94.3%)	0.008
	No	80 (4.1%)	89 (5.3%)	151 (4.6%)	85 (3.8%)	201 (5.5%)	
	Unknown		1 (0.1%)	1 (0.0%)	4 (0.2%)	7 (0.2%)	
Radiation	Yes	1,037 (53.0%)	810 (48.0%)	1,653 (50.6%)	1,116 (50.2%)	1,656 (45.7%)	<0.001
	No	911 (46.6%)	869 (51.5%)	1,603 (49.1%)	1,092 (49.1%)	1,948 (53.7%)	
	Total	8 (0.4%)	10 (0.6%)	8 (0.2%)	17 (0.8%)	21 (0.6%)	
Chemo therapy	Yes	1,481 (75.7%)	1,237 (73.2%)	2,482 (76.0%)	1,659 (74.6%)	2,716 (74.9%)	0.244
	None/Unknown	475 (24.3%)	452 (26.8%)	782 (24.0%)	566 (25.4%)	909 (25.1%)	
Subtype	HER2+	1,956 (100.0%)	1,689 (100.0%)	3,264 (100.0%)	2,225 (100.0%)	3,625 (100.0%)	

Table 4: NDI- Neighborhood deprivation index, Avg: Average, N: Number, HR+ : Hormone receptor-positive human epidermal growth factor 2- negative, HER2 + : Human epidermal growth factor receptor 2 positive

**Table 5:** Survival rates in early-stage overall cohort and breast cancer subtypes by NDI: overall survival and disease-specific survival

NDI Quintiles	5-yr Survival Rate (95% CI)	Median Follow-up (months) (Range)	Log Rank P-value
Overall Cohort			
Overall Survival			
Total	0.87 (0.87, 0.87)	44.0 (0.0, 83.0)	p<0.001
Least Deprivation	0.89 (0.88, 0.89)	54.0 (0.0, 83.0)	
Below Avg Deprivation	0.87 (0.87, 0.88)	53.0 (0.0, 83.0)	
Average Deprivation	0.86 (0.85, 0.86)	46.0 (0.0, 83.0)	
Above Avg Deprivation	0.84 (0.83, 0.85)	49.0 (0.0, 83.0)	
Most Deprivation	0.85 (0.85, 0.86)	49.0 (0.0, 83.0)	
Disease-specific survival			
Total	0.93 (0.93, 0.93)	42.0 (0.0, 83.0)	p<0.001
Least Deprivation	0.94 (0.94, 0.94)	52.0 (0.0, 83.0)	
Below Avg Deprivation	0.93 (0.93, 0.94)	50.0 (0.0, 83.0)	
Average Deprivation	0.92 (0.92, 0.93)	44.0 (0.0, 83.0)	
Above Avg Deprivation	0.91 (0.90, 0.92)	46.0 (0.0, 83.0)	
Most Deprivation	0.92 (0.91, 0.92)	46.0 (0.0, 83.0)	
Triple-negative breast cancer			
Overall Survival			
Total	0.76 (0.75, 0.77)	45.0 (0.0, 83.0)	p= 0.004
Least Deprivation	0.78 (0.75, 0.80)	56.0 (0.0, 83.0)	
Below Avg Deprivation	0.79 (0.76, 0.82)	53.0 (0.0, 83.0)	
Average Deprivation	0.76 (0.73, 0.77)	48.0 (0.0, 83.0)	
Above Avg Deprivation	0.74 (0.71, 0.76)	50.0 (0.0, 83.0)	
Most Deprivation	0.75 (0.73, 0.76)	49.0 (0.0, 83.0)	
Disease-specific survival			
Total	0.81 (0.81, 0.82)	43.0 (0.0, 83.0)	p= 0.007
Least Deprivation	0.83 (0.81, 0.86)	53.0 (0.0, 83.0)	
Below Avg Deprivation	0.85 (0.82, 0.87)	50.0 (0.0, 83.0)	
Average Deprivation	0.82 (0.80, 0.83)	46.0 (0.0, 83.0)	
Above Avg Deprivation	0.80 (0.78, 0.82)	47.0 (0.0, 83.0)	
Most Deprivation	0.81 (0.79, 0.82)	46.0 (0.0, 83.0)	
Hormone-receptor positive breast cancer			
Overall Survival			
Total	0.90 (0.89, 0.90)	43.0 (0.0, 83.0)	p<0.001
Least Deprivation	0.91 (0.90, 0.92)	52.0 (0.0, 83.0)	
Below Avg Deprivation	0.90 (0.89, 0.90)	51.0 (0.0, 83.0)	
Average Deprivation	0.88 (0.87, 0.89)	45.0 (0.0, 83.0)	
Above Avg Deprivation	0.87 (0.87, 0.88)	48.0 (0.0, 83.0)	
Most Deprivation	0.88 (0.88, 0.89)	47.0 (0.0, 83.0)	
Disease-specific survival			
Total	0.96 (0.95, 0.96)	42.0 (0.0, 83.0)	p<0.001
Least Deprivation	0.96 (0.96, 0.97)	50.0 (0.0, 83.0)	
Below Avg Deprivation	0.96 (0.95, 0.96)	49.0 (0.0, 83.0)	
Average Deprivation	0.95 (0.95, 0.96)	43.0 (0.0, 83.0)	
Above Avg Deprivation	0.95 (0.94, 0.95)	46.0 (0.0, 83.0)	
Most Deprivation	0.95 (0.94, 0.95)	45.0 (0.0, 83.0)	
HER2- positive breast cancer			
Overall Survival			
Total	0.87 (0.87, 0.88)	42.0 (0.0, 83.0)	p <0.001
Least Deprivation	0.89 (0.88, 0.91)	50.0 (0.0, 83.0)	
Below Avg Deprivation	0.89 (0.87, 0.91)	49.0 (0.0, 83.0)	
Average Deprivation	0.86 (0.84, 0.87)	43.0 (0.0, 83.0)	
Above Avg Deprivation	0.84 (0.82, 0.86)	44.0 (0.0, 83.0)	
Most Deprivation	0.85 (0.84, 0.87)	46.0 (0.0, 83.0)	
Disease-specific survival			
Total	0.92 (0.91, 0.92)	40.0 (0.0, 83.0)	p <0.001
Least Deprivation	0.93 (0.92, 0.94)	49.0 (0.0, 83.0)	
Below Avg Deprivation	0.93 (0.91, 0.94)	47.0 (0.0, 83.0)	
Average Deprivation	0.91 (0.89, 0.92)	42.0 (0.0, 83.0)	
Above Avg Deprivation	0.89 (0.87, 0.91)	42.0 (0.0, 83.0)	
Most Deprivation	0.90 (0.89, 0.91)	44.0 (0.0, 83.0)	

Table 5: NDI- Neighborhood deprivation index, CI- Confidence Interval, HER2 + : Human epidermal growth factor receptor 2 positive

## Data Availability

All data utilized in this article is available in public datasets such as SEER and NCI Neighborhood deprivation index. Data analyzed during this study are included in this published article and its Appendix.
